# Enhancing the sustainability of cultural identity in science curricula through artificial intelligence as an innovative educational approach

**DOI:** 10.1371/journal.pone.0353777

**Published:** 2026-07-21

**Authors:** Jawaher Alrashood, Omayya Al-Hassan, Sarah Alotaibi, Tahani Alrosaa, Rommel AlAli, Ali Al-Barakat, Abdelrahim Ismail, Yusra Aboud, Ali Abdullatif, Ashraf Zaher

**Affiliations:** 1 Department of Teaching and Learning, College of Education and Human Development, Princess Nourah Bint Abdulrahman University, Riyadh, Saudi Arabia; 2 Department of Psychological Sciences, College of Education, Qatar University, Doha, Qatar; 3 The National Research Center for Giftedness and Creativity, King Faisal University, Al-Ahsa, Saudi Arabia; 4 Department of Education, University of Sharjah, Sharjah, United Arab Emirates; 5 Faculty of Educational Sciences, Yarmouk University, Irbid, Jordan; 6 Department of Curriculum and Instruction, College of Education, King Faisal University, Al-Ahsa, Saudi Arabia; 7 The National Research Center for Giftedness and Creativity, King Faisal University, Al-Ahsa, Saudi Arabia; 8 Department of Arabic Language, College of Arts, King Faisal University, Al Ahsa, Saudi Arabia; 9 Translation, Authorship and Publication Center, King Faisal University, Al-Ahsa, Saudi Arabia; GrandEdu Research School, GERMANY

## Abstract

This study investigates the role of AI as an innovative educational approach in promoting the sustainability of cultural identity within science curricula. To achieve this aim, a qualitative research design was employed, using semi-structured interviews and classroom observations as primary data collection methods. The study sample consisted of forty-seven science teachers from the Al-Qassim and Al-Ahsa regions in Saudi Arabia, selected purposively based on their experience with technology-enhanced instruction. The collected data were analyzed using systematic qualitative procedures involving open and axial coding, which facilitated the emergence of key categories related to AI integration, cultural relevance, and instructional practices. Accordingly, the analytical process is best understood as an inductive thematic qualitative approach rather than a full grounded theory methodology, as it does not include theoretical sampling, data saturation, or iterative cycles of data collection. This clarification ensures methodological transparency and alignment between design and analysis. Findings derived from both teachers’ perceptions and classroom observations indicate that AI is not perceived merely as a tool for knowledge transmission, but rather as a supportive instructional mechanism that enables the contextualization of scientific concepts within local cultural settings. In addition, AI-supported instructional practices appear to foster more interactive and collaborative classroom environments, while also contributing to more responsive approaches to assessment. However, it is important to emphasize that these findings are based on teachers’ reported perceptions and observed instructional practices rather than direct measurement of student learning outcomes. Despite this limitation, classroom observations suggest that integrating culturally relevant examples, analyzing cultural texts, and designing collaborative, culturally responsive learning activities may enhance student engagement and strengthen their connection to cultural identity within science learning contexts. Overall, the study highlights the potential of AI to bridge scientific knowledge and local cultural heritage in science education. It contributes to the growing body of literature on AI in education by emphasizing its role in fostering culturally responsive pedagogy and enhancing meaningful learning experiences. The study further underscores the importance of providing teachers with targeted professional development in AI integration, developing culturally responsive instructional materials, and designing assessment practices that address both conceptual understanding and cultural dimensions of learning.

## 1. Introduction

Cultural identity has become an increasingly important dimension within contemporary educational systems, as it enables learners to develop an understanding of their cultural heritage, community values, and social environment while simultaneously connecting scientific knowledge to meaningful cultural contexts [1,2]. In science education, addressing cultural identity is particularly significant because it enhances students’ awareness of the relationship between science, culture, and society [1,3]. Consequently, the goals of science education have evolved beyond the mere transmission of scientific theories and concepts [4]. Contemporary science curricula increasingly emphasize the development of higher-order thinking skills, critical reflection, and students’ ability to interpret scientific knowledge in relation to their surrounding cultural and environmental contexts. Moreover, integrating cultural and social values into science curricula strengthens the relevance of education to students’ lived experiences and supports their sense of belonging and identity [2–4].

From this perspective, cultural identity contributes to the preparation of socially aware and culturally responsive learners who are capable of engaging with future societal challenges [4]. Students who learn science through culturally meaningful contexts are more likely to become active participants in the learning process because they perceive scientific knowledge not as isolated academic content, but as a means of understanding their communities and environments [3,5]. Furthermore, strengthening cultural identity promotes students’ sense of pride and belonging to their communities while encouraging them to preserve and sustain local cultural practices and values. This orientation supports the development of sustainable science curricula that integrate contemporary scientific knowledge with local cultural dimensions [5–7].

In parallel with these educational transformations, Artificial Intelligence (AI) has emerged as a powerful technological innovation with the potential to reshape instructional design and classroom practices across educational disciplines [8,9]. Educational technologies have evolved significantly from merely facilitating access to information toward supporting interactive, adaptive, and personalized learning experiences. Current AI-supported tools can assist in analyzing educational texts, designing engaging instructional activities, generating adaptive learning pathways, and supporting intelligent tutoring systems that respond to students’ individual learning needs [9,10]. In addition, AI technologies can assist teachers in developing instructional strategies that take into account students’ cultural and social backgrounds, thereby supporting inclusiveness and enhancing learning experiences [10,11].

Despite the growing body of research examining AI in education, most prior studies [12–16] have primarily focused on technical, cognitive, and performance-related dimensions, with comparatively limited attention given to the role of AI in supporting cultural identity and contextualized learning within students’ sociocultural environments. Nevertheless, a number of recent studies [13–15] have begun to highlight the potential of AI in preserving cultural heritage, analyzing cultural artifacts, and integrating culturally relevant resources into educational settings. These studies suggest that AI can contribute to culturally responsive pedagogy, particularly when the design and implementation of AI technologies are aligned with the linguistic, social, and cultural characteristics of the educational context [17–19].

Responding to this research gap, the current study seeks to investigate the potential of AI technologies as an innovative pedagogical approach for enhancing the sustainability of cultural identity in science education. Specifically, the study explores how generative AI tools, including ChatGPT and DALL·E, can support the transformation of local cultural resources and science-related content into instructional materials, learning activities, and formative assessment tasks [17,18]. In practice, ChatGPT was utilized to generate culturally contextualized explanations of scientific concepts, develop inquiry-based classroom activities connected to local cultural practices, and support teachers in creating discussion prompts that link scientific ideas to students’ lived experiences. Similarly, DALL·E was used to generate visual representations of scientific phenomena embedded within culturally familiar contexts, thereby supporting visual and culturally responsive learning experiences in science classrooms [18,19].

To explore these issues in depth, the study adopts a qualitative methodology based on semi-structured interviews and classroom observations to document teachers’ perceptions and classroom practices regarding AI integration in science education. The analytical framework is organized around three interrelated dimensions: (1) AI-supported pedagogical practices in science teaching, (2) the integration of cultural identity within science learning, and (3) the classroom implementation of AI-generated instructional resources. The findings suggest that AI integration extends beyond cognitive support and technological facilitation to include culturally situated learning experiences, contextualization of scientific knowledge within local environments, and reinforcement of teachers’ roles as mediators between scientific understanding and cultural identity. Based on these considerations, the study addresses the following central research question: *How can Artificial Intelligence contribute as an innovative pedagogical approach to enhancing the sustainability of cultural identity in science education curricula?*

## 2. Theoretical literature

Recognizing and strengthening cultural identity plays a central role in shaping learners’ personalities, reinforcing their sense of belonging, and developing their social and moral value systems [20,21]. Educational research consistently demonstrates that learning becomes less meaningful when knowledge is presented as value-free information disconnected from learners’ cultural and social realities. Consequently, contemporary educational approaches increasingly emphasize the holistic development of learners’ cognitive, affective, and behavioral dimensions through the integration of cultural values and social norms into educational experiences [21–23]. Such integration enables learners to interpret scientific knowledge within the context of their lived experiences and social environments, thereby promoting deeper engagement with learning.

In line with this perspective, recent educational trends [4,7,14,16] stress the importance of designing curricula and educational programs that reflect the cultural specificities of communities while simultaneously supporting the sustainability of cultural identity in the face of globalization and rapid technological change. Culturally responsive curricula not only strengthen social cohesion, but also develop learners’ capacity to interact constructively within their communities while appreciating cultural diversity and understanding other societies critically and respectfully [21–23]. This balanced educational orientation contributes to the development of socially, culturally, and intellectually aware learners capable of engaging effectively in contemporary society.

Within science education, the relationship between cultural identity and curriculum design is particularly significant because science curricula are closely connected to issues related to students’ daily lives, environmental awareness, health, social relationships, and patterns of production and consumption [24–26]. The applied nature of science education creates considerable opportunities to connect scientific concepts with local cultural contexts, especially through environmental and community-based issues. Accordingly, when science education incorporates culturally relevant practices and examples, learning becomes more meaningful and encourages learners to think responsibly about social and environmental challenges, thereby contributing to sustainable educational outcomes [23,24,26].

Previous studies [26–28] further indicate that embedding cultural identity into science education supports meaningful learning by enabling students to understand scientific concepts through contexts rooted in their own culture and society. Connecting scientific knowledge with learners’ environments and daily experiences enhances conceptual understanding, reduces passive learning, and increases students’ willingness to actively participate in the educational process [27,29]. Moreover, this approach promotes critical and analytical thinking by encouraging learners to draw upon their cultural experiences and prior knowledge to interpret scientific concepts and engage scientifically with traditional practices and local phenomena [28,30,31].

Consistent with these findings, several studies [29–33] suggest that integrating cultural identity into science education strengthens the relationship between scientific knowledge and learners’ social and cultural identities. This integration contributes to presenting science as a socially meaningful and culturally connected form of knowledge rather than merely an abstract academic discipline [33,34]. Furthermore, culturally integrated science education reflects the broader principles of education for sustainability by equipping learners with the scientific knowledge, cultural awareness, and social skills necessary to engage responsibly with emerging scientific and technological developments while preserving local cultural values and traditions [34–36].

Alongside these educational developments, Artificial Intelligence (AI) has emerged as a transformative technological innovation capable of reshaping educational practices and enhancing science learning experiences [37–39]. AI technologies are increasingly being integrated into educational systems to support scientific inquiry, facilitate conceptual understanding, and improve the teaching and learning of scientific phenomena [39,40]. Through AI-supported applications, digital simulations, and interactive models, learners can better understand scientific concepts while simultaneously connecting these concepts to their cultural backgrounds and environmental realities [11,4–44]. As a result, AI has the potential to make science learning more relevant to students’ social and cultural contexts.

In this regard, Tassoti [45] highlighted the role of AI in enhancing science curricula through adaptive learning systems capable of presenting scientific concepts according to learners’ cognitive levels and cultural backgrounds. AI-supported educational analytics also provide teachers with opportunities to better understand students’ interactions with scientific activities in relation to their cultural knowledge and experiences. Such applications can help identify misconceptions, personalize instruction, and encourage students to explore scientific concepts through culturally meaningful learning pathways [39,40]. Furthermore, AI-supported interactive activities that simulate students’ physical and social environments can create learning experiences that are simultaneously scientifically rigorous and culturally responsive [44,45].

Research also suggests that culturally responsive AI design positively influences learners’ motivation and engagement in science education [36,37]. Integrating scientific learning with cultural experiences enables students to interpret scientific phenomena more comprehensively while enhancing higher-order thinking, critical analysis, and problem-solving skills without neglecting the cultural and social dimensions of learning [15,16,45]. This integration supports learners’ ability to engage with scientific advancements while maintaining meaningful connections to culturally valued local practices and traditions.

Similarly, AlAli and Al-Barakat [46] emphasized that AI can support educators in designing culturally responsive curricula and learning activities that combine data-driven instructional practices with culturally informed educational experiences. Such approaches contribute to the creation of inclusive learning environments that strengthen both scientific understanding and students’ social and cultural sense of belonging [15,46]. In this context, AI-supported education aligns closely with the goals of sustainable education by preparing learners with scientific knowledge and culturally grounded competencies necessary for addressing contemporary environmental and societal challenges [38,39].

Likewise, Al-Barakat et al. [15] argued that AI facilitates locally responsive education when integrated within pedagogical approaches designed to address learners’ linguistic and cultural needs. This includes contextualizing instructional materials through local examples, designing activities that encourage students to draw upon their cultural experiences in interpreting scientific concepts, and adapting learning resources to individual learner differences. Such practices increase learner engagement, bridge gaps between scientific knowledge and lived experience, and enhance the relevance of science education to students’ immediate social and cultural environments [47,48].

Despite these promising developments, numerous researchers [17–20,37,39,47–50] argue that most existing studies on AI in education continue to focus primarily on technical efficiency, academic achievement, cognitive outcomes, and higher-order thinking skills, while comparatively neglecting the cultural dimensions of AI-supported learning. Similarly, several studies [7,9,11,23,27,51–54] continue to treat culture as a separate component rather than as an integrated dimension within science curricula. This separation limits opportunities to meaningfully connect scientific learning with learners’ cultural identities and local contexts. Consequently, integrating AI into science curricula from a culturally responsive perspective may provide opportunities to promote meaningful learning, enhance higher-level analytical and reflective thinking, and strengthen learners’ cultural and social belonging [50–53,55,56].

Overall, the reviewed literature reveals a limited number of studies that attempt to integrate AI, science education, and cultural identity within a unified pedagogical framework, particularly within Arab educational contexts. The literature also highlights the pivotal role of teachers as cultural mediators who employ AI technologies to support sustainable education and design balanced curricula that integrate scientific advancement with learners’ cultural identities. Such approaches can contribute to preparing students to engage thoughtfully and responsibly with contemporary global and societal challenges while maintaining meaningful connections to their cultural heritage.

## 3. Method

### 3.1 Research design

This research aimed to examine the integration of artificial intelligence and its impact on the preservation and sustainability of learners’ cultural identity within science pedagogy. Given that the study investigates a complex educational phenomenon embedded in real classroom contexts, where meanings, perceptions, and practices are central, a qualitative research design was adopted as the most appropriate approach. This choice was made because the study seeks to understand *how* and *why* AI is integrated into teaching practices and how it is perceived to influence cultural identity, rather than to measure variables or test predefined hypotheses.

Unlike quantitative approaches, which focus on numerical measurement and statistical generalization, this study required an in-depth exploration of teachers’ experiences and classroom interactions in natural settings. Similarly, a mixed-methods design was not adopted because the primary aim was not to combine statistical generalization with qualitative explanation, but rather to generate rich, contextualized insights into pedagogical practices and cultural meanings associated with AI integration in science education.

**Fig 1:**
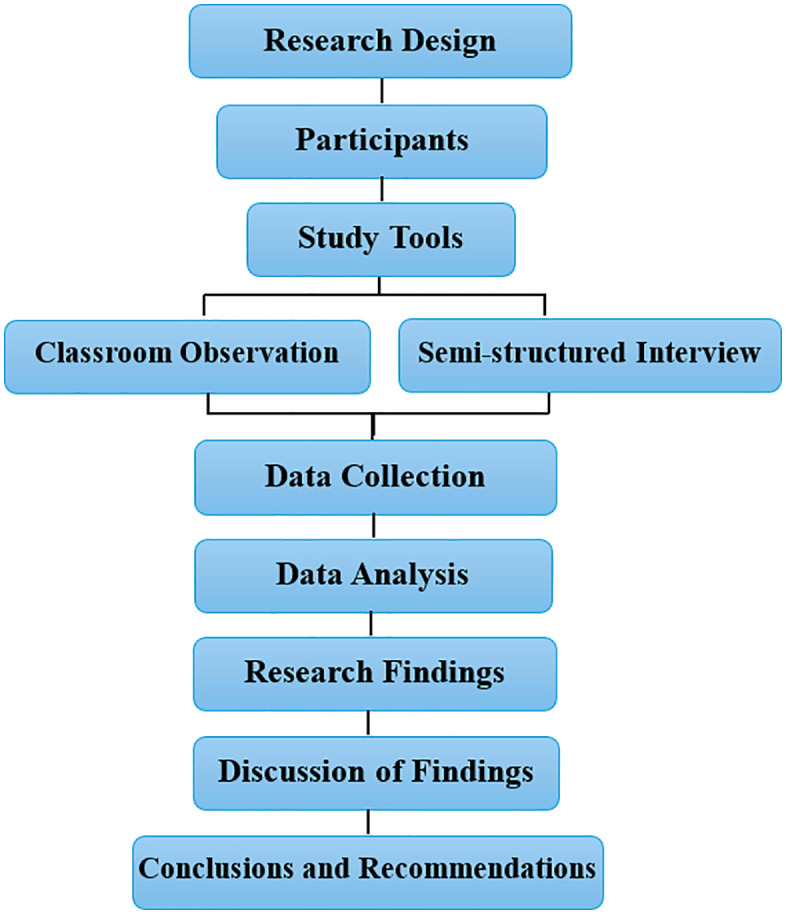
Research design.

To achieve this purpose, the researchers employed a combination of direct classroom observations and semi-structured interviews. Classroom observations allowed for the documentation of actual instructional practices and the ways in which AI was integrated to support cultural identity in authentic learning environments. Semi-structured interviews were then used to further explore teachers’ interpretations of these observed practices and to gain deeper insights into their pedagogical reasoning and experiences. Overall, the qualitative design was deemed most suitable for capturing the complexity, contextuality, and meaning-making processes associated with AI integration in real classroom settings. More specifically, the study design is represented as shown in Fig 1.

Fig 1 illustrates the systematic qualitative data analysis approach based on sequential and interconnected stages, beginning with data collection and organization, followed by coding, categorization, thematic analysis, and interpretation. This process culminated in the development of a conceptual framework illustrating Artificial Intelligence as an Innovative Instructional Approach for Enhancing the Sustainability of Cultural Identity within Science Curricula.

### 3.2 Participants

This study employed *a* purposive sampling strategy facilitated through convenience access *procedures* to investigate the role of artificial intelligence as an emerging educational tool in promoting the sustainability of cultural identity within the science curriculum. The purposive aspect of the sampling process involved selecting participants according to specific professional criteria aligned with the objectives of the study. Participants were required to be science teachers working in private primary schools, possess direct experience in science instruction, and demonstrate an interest in or experience with the use of contemporary educational technologies, including AI-supported teaching practices. At the same time, convenience considerations influenced participant recruitment, as the researchers selected schools and teachers that were accessible and willing to participate during the data collection period. This approach ensured the inclusion of participants capable of providing rich and relevant insights while also enabling practical access to the research field.

The study sample consisted of 47 male and female science teachers from private primary schools located in the Al-Ahsa and Al-Qassim regions of the Kingdom of Saudi Arabia. Selecting participants from these two regions enabled the study to examine the integration of artificial intelligence in science education within educational environments that share similar cultural and social characteristics while also reflecting a degree of contextual diversity. This contributed to enriching the findings without compromising the shared national cultural framework. In terms of gender distribution, the sample included 27 female teachers (57.4%) and 20 male teachers (42.6%), thereby allowing the collection of diverse perspectives regarding teaching practices, approaches to integrating AI into the science curriculum, and the perceived influence of such practices on preserving students’ cultural identity.

The sample also reflected an appropriate level of academic and professional diversity relevant to the study objectives. Twenty-nine participants (61.7%) held a bachelor’s degree in science accompanied by a teaching qualification, 12 participants (25.5%) held a master’s degree in science education, and 6 participants (12.8%) held a doctorate in science education or curriculum development. In addition, the participants represented a broad range of teaching experience*,* with 10 teachers (21.3%) having fewer than five years of experience, 17 teachers (36.2%) having between five and less than ten years of experience, and 20 teachers (42.5%) having more than ten years of teaching experience. This diversity strengthened the study by incorporating the perspectives of both novice teachers, who may be more receptive to digital innovation, and experienced teachers, who possess deeper insights into culturally responsive science teaching and the sustainability of cultural identity within educational contexts.

All participants worked in educational settings characterized by relatively similar organizational and pedagogical structures, including comparable technological infrastructure, access to modern digital teaching tools, class sizes, and flexibility in curriculum implementation and instructional methods. Such similarities helped minimize the influence of external contextual variables and allowed the study to focus more specifically on the role of artificial intelligence in sustaining cultural identity within the science curriculum. Furthermore, these educational environments provided opportunities for implementing innovative instructional approaches that integrate scientific concepts with local cultural values, thereby strengthening students’ cultural identity while aligning with contemporary trends in science education.

### 3.3 Data collection through classroom observation

The primary method of data collection in this study was *classroom observation*, designed to investigate the use of artificial intelligence in fostering the cultural identity of learners within primary-level science classrooms. Classroom observations were systematically and rigorously conducted during science lessons for Grades 6–9, with each session lasting 45 minutes. These in-class observations provided the researchers with a comprehensive understanding of instructional strategies, teacher–student interactions, and the integration of artificial intelligence into teaching and learning processes. All observations were conducted over the period from 25 August 2025–30 October 2025, spanning two months and five days, ensuring systematic and sustained data collection.

The observation indicators were designed to capture multiple dimensions relevant to the study, including teaching materials and technologies employed, teacher–student interaction patterns, the stimulation of critical and creative thinking, and the level of student engagement in classroom activities. Particular attention was given to the ways in which artificial intelligence was used to enhance learners’ cultural identity and to connect scientific content with local community and cultural contexts*.* The classroom observation checklist used in this study was developed based on relevant literature and was reviewed and validated by specialists in science education and qualitative research. The final version of the checklist is provided as supplementary material (S1 Appendix), ensuring transparency, validity, and replicability of the research instrument.

The observations were conducted by two trained observers who were qualified educators in science education. Each observer independently used the standardized observation checklist to document classroom practices. To ensure methodological rigor, *inter-rater reliability* was assessed using Cohen’s Kappa coefficient. The level of agreement between the two independent observers was calculated based on observed agreement (Po) and expected agreement by chance (Pe), using the formula κ = (Po − Pe)/ (1 − Pe). The results indicated a very strong level of agreement between the observers (κ = 0.86). Following independent observations, a calibration meeting was conducted to discuss and reconcile any discrepancies and to ensure consistency in interpretation and recording of data.

To enhance credibility and trustworthiness, multiple structured observations were conducted at different times and classroom contexts to reduce the influence of situational bias and to increase the representativeness of the data. In addition, observations were supported by detailed field notes written during each session, capturing contextual information such as classroom environment, instructional focus, and the specific ways in which AI tools were utilized to support cultural identity within science teaching.

### 3.4 Semi-structured interviews

To address the objective of examining the role of Artificial Intelligence (AI) as an innovative pedagogical approach in promoting the sustainability of cultural identity within science curricula, the researchers employed individual semi-structured interviews as a primary qualitative data collection method. This approach provided sufficient flexibility to adapt interview questions according to emerging themes during the interview process, thereby enabling a rich and in-depth exploration of the phenomenon under investigation in alignment with qualitative research principles.

The interviews were conducted with the same science teachers who participated in the classroom observations, ensuring methodological triangulation across data collection methods. Classroom observations were conducted first, followed by semi-structured interviews with the same participants, and both procedures were completed within the same data collection period, extending from 25 August 2025–30 October 2025. After obtaining written informed consent, all interviews were audio-recorded in accordance with established ethical research guidelines and conducted in quiet locations within the school premises. Each interview lasted approximately between 45 and 55 minutes.

The interview guide was developed based on the study’s theoretical framework and relevant literature related to science education, Artificial Intelligence in education, and the sustainability of cultural identity. The interviews primarily focused on exploring teachers’ perceptions, experiences, and instructional practices concerning the integration of AI within science curricula. Accordingly, the interview questions were designed as open-ended and exploratory to encourage detailed reflection and discussion (See S2 Appendix), as presented below:

1. How do teachers understand the concept of sustaining cultural identity in the context of AI-enhanced science instruction?
2. How does the use of artificial intelligence help link scientific concepts with local cultural and environmental values?
3. What instructional strategies do teachers employ to integrate AI in ways that foster cultural belonging and personalized learning among students?
4. How are AI tools incorporated into classroom or extracurricular activities to support cultural values and sustainable behavior?
5. How do teachers evaluate the role of the school in supporting the use of AI to promote the sustainability of cultural identity within science curricula?

To ensure content validity, the interview guide was reviewed by a panel of eleven experts in educational technology, artificial intelligence, science education, curriculum studies, and educational research. Their feedback was incorporated to improve clarity, specificity, and contextual relevance. A pilot study was then conducted with nine teachers outside the main sample to refine wording and ensure suitability within the local educational context.

For data analysis, interview transcripts were independently analyzed by two researchers. The unit of analysis consisted of meaning units (sentences or paragraphs conveying a single idea related to AI integration, teaching practices, or cultural identity sustainability), which were extracted from the transcribed interviews. These meaning units were then systematically compared across both coders to ensure consistency in interpretation and coding. Inter-rater reliability was calculated using Cooper’s percentage agreement formula (Agreement % = Number of Agreements/ Total Number of Coding Decisions × 100), where coding decisions referred to the assignment of meaning units to analytical categories. The analysis yielded a high agreement rate of 95%, indicating strong consistency between the two researchers.

Overall, the combined use of semi-structured interviews and classroom observations provided a comprehensive understanding of how artificial intelligence is integrated into science curricula as an innovative pedagogical approach that supports the sustainability of students’ cultural identity, thereby contributing robust empirical evidence to address the study’s research objectives.

### 3.5 Data analysis

The study adopted a qualitative analytical framework informed by grounded theory principles and implemented through qualitative content analysis, with the aim of understanding the role of Artificial Intelligence (AI) as an innovative educational tool in fostering Cultural Identity Sustainability (CIS) within an integrated science curriculum. This framework provided the overarching approach for analyzing data derived from classroom observations and semi-structured interviews, guiding the systematic progression from raw data to scientific interpretation. Grounded theory principles informed the iterative processes of coding, categorization, and theme development, while qualitative content analysis served as the primary interpretive method for examining instructional practices and classroom interactions involving AI-supported technologies (See S3 Appendix). This integrated approach enabled the identification of recurring pedagogical patterns, cultural representations, and instructional practices within science education contexts.

Building on this foundation, data analysis proceeded through continuous and iterative cycles of data collection and review to ensure a progressively deeper and more refined understanding of the phenomena under investigation. This stage constituted a preliminary phase for developing initial conceptual insights from the data. Open coding procedures, derived from grounded theory, were employed to generate initial meaning units directly from the raw data. These units captured multiple dimensions, including teachers’ instructional practices, students’ classroom interaction patterns, the use of AI-based educational tools, and culturally related values and identity constructs relevant to the study context.

Subsequently, the constant comparative method was applied to systematically compare codes and data segments in order to refine categories and establish meaningful relationships among them. This iterative process facilitated the transition from descriptive coding to higher-level interpretive analysis, allowing the researchers to reorganize related meaning units and identify conceptual linkages. As a result, the data were classified into broader analytical themes reflecting the interconnected pedagogical, cultural, and technological dimensions of the study.

As these categories were further refined, qualitative content analysis was used to interpret and contextualize their meanings in relation to the role of AI in science education and its contribution to sustaining cultural identity. The integration of grounded theory with content analysis enabled a multi-layered analytical perspective linking classroom practices with broader cultural and technological contexts.

To ensure methodological transparency, it is important to note that the frequencies and percentages reported in this study represent the number of teachers in whom each observed practice was identified at least once during the observation period, rather than the frequency of occurrences within a single lesson. Similarly, interview-derived values reflect the number of participants who expressed a given idea or concept at least once across interviews, thereby indicating the prevalence of the concept among participants rather than its repetition within individual interviews.

Finally, based on this integrated analytical procedure, the findings from classroom observations and interviews were synthesized into six interrelated dimensions representing the overall structure of the phenomenon under investigation:

1. AI-supported personalization of educational content
2. Analysis of cultural texts and sources
3. Designing AI-supported interactive learning activities
4. Utilizing artificial intelligence in curriculum development
5. Promoting interactive and collaborative learning
6. Intelligent assessment of cultural values in learning

**Fig 2:**
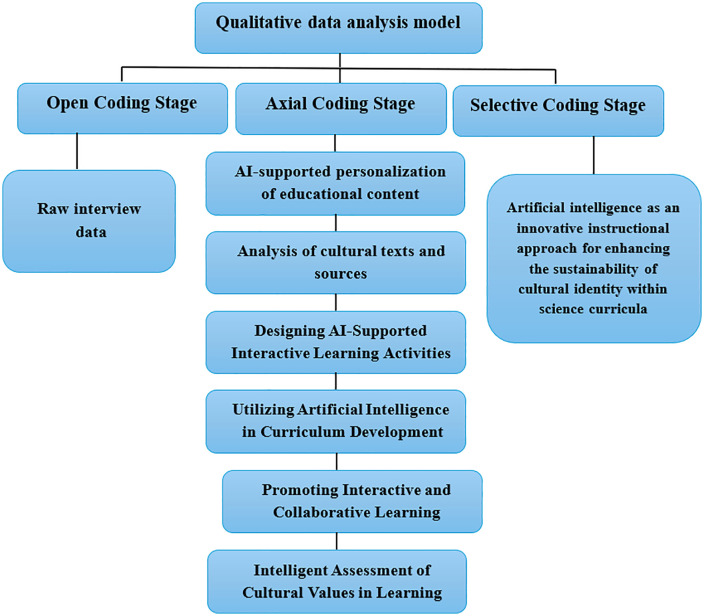
Semi-structured analysis model.

In light of this analytical sequence, the conceptual framework of the study’s data analysis is illustrated in Fig 2.

### 3.6 Ethical considerations

Ethical considerations were carefully addressed throughout all stages of the study, including research design, data collection, analysis, and reporting. The study involved science teachers who participated in classroom observations and semi-structured interviews, and all collected data were treated confidentially and used solely for research purposes. Ethical approval was obtained from the Research Ethics Committee at King Faisal University under Decision No. KFU-REC-2025JUN-EA000985 (12 June 2025), in accordance with local and international ethical standards. Prior to data collection, participants were informed about the study’s aims, procedures, and their rights, including voluntary participation and the right to withdraw at any stage without consequences. Written informed consent was obtained from all participants before conducting classroom observations and interviews. To ensure anonymity and confidentiality, pseudonyms were used during analysis and reporting, and all identifying information related to participants and schools was removed. All data were securely stored and made accessible only to the research team.

## 4. Results of the study

The primary research question examined the role of artificial intelligence as an innovative educational approach for enhancing the sustainability of cultural identity within science education curricula. To address this objective, qualitative data were collected through classroom observations and semi-structured interviews with science teachers. The study revealed the use of different forms of artificial intelligence tools, including generative AI systems, adaptive learning technologies, simulation-based applications, and intelligent tutoring systems. These tools were mainly applied by teachers to support lesson preparation, personalize instructional content, generate culturally relevant scientific examples, and design interactive learning and assessment activities within classroom practice. In practice, AI-generated outputs included context-based scientific scenarios, culturally adapted explanations, interactive questions, and formative assessment tasks aligned with students’ everyday and cultural experiences, reflecting the integration of AI into both instructional design and classroom implementation (See S4 Appendix).

Building on these applications, the study further explored how generative AI tools such as ChatGPT and DALL·E were utilized to transform local cultural resources and science-related content into instructional materials, learning activities, and formative assessment tasks. ChatGPT was used to generate culturally contextualized explanations of science concepts, design inquiry-based classroom activities linked to local cultural practices, and support teachers in developing discussion prompts that connect scientific ideas with students’ lived experiences. This process enabled teachers to move from general instructional planning toward more culturally responsive and context-sensitive teaching practices. Similarly, DALL·E was used to generate visual representations of scientific phenomena embedded within local cultural contexts; these visuals were incorporated into teaching materials to enhance conceptual clarity, engagement, and visual understanding in science classrooms. Collectively, these AI-supported practices informed the structure of the analysis, leading to the organization of findings into the following analytical themes:

1. AI-supported personalization of educational content
2. Analysis of Cultural Texts and Sources
3. Designing AI-Supported Interactive Learning Activities
4. Utilizing Artificial Intelligence in Curriculum Development
5. Promoting Interactive and Collaborative Learning
6. Intelligent Assessment of Cultural Values in Learning

The findings related to each theme are presented below.

### 4.1 Results of Theme One: AI-supported personalization of educational content

Classroom observations revealed that most science teachers (46 out of 47; 97.9%) utilized artificial intelligence tools to integrate local cultural elements into science instruction and connect scientific concepts with students’ lived experiences. Teachers frequently adapted scientific examples and classroom activities to culturally familiar contexts, such as desert climate conditions, agriculture, and water conservation practices, thereby increasing the relevance and accessibility of scientific content. These practices appeared to enhance students’ engagement and strengthen the connection between scientific understanding and cultural identity. Table 1 summarizes the main observed teaching practices associated with AI-supported personalization of educational content and culturally responsive science instruction.

**Table 1:** Classroom observation analysis of AI-supported personalization of educational content.

No.	Learning Practices	Frequency	Percentage (%)
1	Integrating physics concepts with the local environment	46	97.9
2	Linking lessons to familiar cultural practices	45	95.7
3	Adapting examples to students’ varying levels	44	93.6
4	Using AI tools to explain scientific experiments	42	89.4
5	Encouraging students to provide local examples	40	85.1

*Note: The reported percentages refer to the number of teachers in which each observed practice was identified at least once during the observation period.*

Table 1 shows that teachers demonstrated consistent efforts to connect students’ lived experiences and cultural knowledge with scientific concepts. These instructional practices supported students’ understanding of scientific concepts in relation to their daily lives and enhanced their engagement in classroom learning activities. The findings further indicate that Artificial Intelligence (AI) was utilized to generate culturally responsive teaching examples, propose culturally relevant scientific experiments, and encourage students to provide examples derived from their own cultural contexts. Collectively, these practices reflect teachers’ awareness of the close relationship between scientific knowledge, cultural context, and students’ cultural identity within science education.

The interview findings further supported these observations. A substantial majority of participants (91%) reported that AI-supported personalization of educational content contributes to sustaining cultural identity by integrating local cultural elements and everyday experiences into science learning. One participant explained:

*“AI tools help me present scientific content in a way that aligns with the Saudi environment. When I connect physics concepts to the desert climate or the nature of the land, the lesson becomes more accessible to students.”*

Another participant emphasized the importance of linking scientific concepts to culturally familiar practices:

*“Being able to use AI-supported intelligent personalization means that I can connect science lessons to readily identifiable practices like farming or water saving, which makes students feel that science is embedded in their culture.”*

A third participant highlighted the influence of localized AI-generated examples on student engagement and understanding:

*“When the intelligent system suggests local examples that match students’ levels, I notice a clear improvement in engagement and comprehension.”*

Overall, the combined findings from classroom observations and interviews suggest that artificial intelligence can support culturally responsive science teaching by connecting scientific concepts to students’ local environments and lived experiences. The findings further indicate that AI may enhance the contextual relevance and meaningfulness of science learning while supporting the preservation of learners’ cultural identity within educational settings.

### 4.2. Results of Theme Two: Analysis of cultural texts and sources

Classroom observations indicated that a large proportion of science teachers (40 out of 47; 85.1%) used artificial intelligence tools to incorporate cultural texts and local cultural sources into science instruction. Teachers employed AI to identify and integrate culturally relevant materials, such as heritage stories, traditional proverbs, and local customary practice, when explaining scientific concepts. These practices appeared to support students’ cultural identity and enhance the contextual relevance of science learning. Table 2 presents the observed classroom practices related to the use of artificial intelligence in analyzing and integrating cultural texts and sources within science teaching.

**Table 2:** Classroom observation analysis of cultural texts and sources.

No.	Instructional Practices	Frequency	Percentage (%)
1	Linking heritage stories to scientific concepts	40	85.1
2	Extracting environmental values from local heritage	38	80.8
3	Integrating traditional proverbs with scientific experiments	37	78.7
4	Analyzing heritage texts to extract scientific principles	36	76.6
5	Enhancing discussions on cultural practices and science	35	74.5

Table 2 indicates that teachers did not present scientific content in an abstract or decontextualized manner; instead, they intentionally integrated scientific concepts with students’ local cultural contexts, thereby enhancing the relevance and meaningfulness of learning. This culturally responsive approach was associated with higher levels of student engagement and motivation and supported deeper conceptual understanding rather than rote memorization.

Findings further show that artificial intelligence served as a supportive pedagogical tool that enabled teachers to identify, extract, and integrate culturally relevant materials, such as heritage narratives, traditional proverbs, and local environmental practices, into science instruction. This facilitated the construction of meaningful connections between scientific concepts and students’ lived cultural experiences, thereby strengthening the cultural relevance of science teaching.

Interview data were consistent with the classroom observations. A majority of participants (85.1%; 40 out of 47) reported that using AI to analyze and integrate cultural texts and sources is an effective pedagogical practice for sustaining cultural identity in science education. One teacher stated:

*“AI assists me in using heritage stories and traditional practices, integrating them with science lessons, and allowing students to perceive the curriculum as culturally relevant to them.”*

Another participant noted:

*“Using AI tools, I can extract environmental values from local heritage and relate them to environmental science lessons, such as conserving natural resources.”*

Similarly, one participant explained:

*“Integrating traditional proverbs or practices with scientific concepts, supported by AI, make learning more meaningful and impactful.”*

Overall, the convergence of observational and interview data suggests that AI-supported analysis of cultural resources can enhance culturally responsive science teaching. This, in turn, may strengthen student engagement, promote deeper learning, and reinforce students’ sense of cultural identity within science education contexts.

### 4.3 Results of Theme Three: Designing AI-supported interactive learning activities

Classroom observations indicated that most participating teachers (42 out of 47; 89.4%) effectively utilized Artificial Intelligence (AI) tools to design interactive learning activities that support students’ cultural identity within science education contexts. These activities were culturally contextualized and included science experiments linked to the local environment, culturally themed competitions, and digital learning games connecting scientific concepts with real-world applications. Overall, AI was employed to strengthen the integration of cultural relevance into interactive science learning and to connect students’ lived experiences with scientific content.

To further illustrate the observed instructional practices related to AI-supported curriculum development, Table 3 presents the frequencies and percentages of the major classroom practices identified during the observation process.

**Table 3:** Classroom observation results on the use of AI in curriculum development.

No.	Instructional Practices	Frequency	Percentage (%)
1	Designing hands-on experiments linked to the local environment	42	89.4
2	Organizing interactive competitions based on cultural elements	40	85.1
3	Using educational games to simplify scientific concepts	39	82.9
4	Encouraging students to suggest heritage-inspired solutions	38	80.8
5	Incorporating interactive technologies into classroom presentations	37	78.7
6	Using culturally grounded digital storytelling	35	74.5
7	Customizing learning activities based on students’ cultural backgrounds	33	70.2

Table 3 indicate that the integration of artificial intelligence in designing interactive learning activities ranged from 70.2% to 89.4% across lessons, suggesting an increasing adoption of intelligent technologies in science teaching practices. Teachers frequently designed hands-on, environment-based activities that connected scientific concepts to students’ everyday experiences, thereby supporting applied scientific understanding. In addition, AI-supported interactive competitions, educational games, and digital storytelling activities were culturally oriented, which contributed to increased student engagement and motivation. These activities also encouraged students to propose scientific solutions grounded in local cultural heritage, thereby strengthening the connection between science learning and cultural identity.

Interview data aligned with these observations, as 89.4% of participants emphasized the importance of AI-supported interactive activities in sustaining cultural identity. One participant stated:

*“AI helps me create local heritage-focused activities and educational activities, which help students engage with scientific concepts.“*

Another participant said:

*“Intelligent tools help me create educational games so students can bridge cultural values and scientific concepts. It’s so much fun.”*

Overall, the findings suggest that AI functions as an enabling pedagogical tool that supports culturally responsive interactive learning in science education. Such practices contribute to more engaging learning environments and appear to enhance students’ cultural belonging, motivation, creativity, and conceptual understanding, thereby improving the overall quality of science education.

### 4.4 Results of Theme Four: Utilizing artificial intelligence in curriculum development

Classroom observations indicated that the majority of participating teachers (87.2%) effectively utilized Artificial Intelligence (AI) tools to support curriculum development practices aimed at sustaining cultural identity within science education. Teachers primarily employed AI to design and adapt instructional materials and learning activities that integrated local cultural values and contexts, including community life, natural resources, and students’ everyday experiences, while maintaining scientific accuracy and age-appropriate content. Overall, AI functioned as a supportive instructional tool for aligning curriculum content with both scientific standards and culturally relevant educational contexts. To provide a clearer overview of the observed practices related to AI-supported curriculum development, Table 4 presents the frequencies and percentages of the major instructional practices identified during classroom observations.

**Table 4:** Classroom observations on the use of AI in curriculum development.

No.	Instructional Practices	Frequency	Percentage (%)
1	Developing instructional units aligned with community context	41	87.2
2	Updating scientific content while preserving cultural relevance	39	82.9
3	Designing learning activities reflecting local traditions	38	80.8
4	Proposing community-related applied topics	37	78.7

Table 4 indicates a consistently high level of AI integration in curriculum development practices, ranging from 76.6% to 87.2%. This reflects the widespread use of artificial intelligence in supporting the design and adaptation of instructional materials that align scientific content with learners’ cultural contexts.

The highest frequency (87.2%) was recorded for the development of instructional units linked to community realities. Teachers used AI to design units addressing contemporary issues such as renewable energy and water conservation, thereby connecting scientific concepts to students’ everyday lives. Other practices included updating scientific content while maintaining cultural relevance (82.9%), embedding cultural identity within learning activities (80.8%), proposing culturally relevant applied topics (78.7%), and designing culturally contextualized assessments (76.6%).

Interview findings were consistent with classroom observations. Most participants (38 out of 47; 81%) reported that AI-supported curriculum development facilitates the integration of updated scientific knowledge with local cultural content. One teacher stated:

*“AI assists in constructing instructional units that serve the sociocultural realities of Saudi Arabia such as renewable energy and water conservation.”*

Another participant noted:

*“AI in curriculum development ensures that information will be up to date, and the cultural dimension will not be lost.”*

A third participant added:

*“When the curriculum culturally represents the society it serves, it has more value and impact for the learners.”*

Overall, the findings suggest that AI serves as an enabling tool for aligning curriculum content, learning activities, and assessment with both scientific accuracy and cultural relevance. This integration enhances student engagement and supports the preservation of cultural identity by connecting scientific learning to learners’ sociocultural contexts.

### 4.5 Results of Theme Five: Promoting interactive and collaborative learning

Classroom observations indicated that most participating science teachers effectively integrated Artificial Intelligence (AI) tools to support interactive and collaborative learning within science classrooms. These instructional practices positioned students as active participants in the construction of knowledge and enabled them to interpret scientific concepts in relation to their everyday experiences and sociocultural contexts.

Teachers employed a variety of AI-supported instructional strategies to enhance classroom interaction, including collaborative learning tasks, interactive science activities, and community-based projects. The diversity of these practices reflects the flexibility of AI technologies in addressing different learning needs and supporting both individual and collaborative learning processes. To further illustrate the observed practices related to interactive and collaborative learning, Table 5 presents the major classroom practices identified during the observation process, together with their corresponding frequencies and percentages.

**Table 5:** Classroom observation for promoting interactive and collaborative learning.

No.	Instructional Practices	Frequency	Percentage (%)
1	Enhancing group and interactive discussions	41	87.2
2	Implementing AI-supported group projects	39	83.0
3	Sharing examples from the local environment	40	85.1
4	Organizing interactive educational competitions	38	80.8
5	Group problem-solving activities	36	76.6

Table 5 shows that participants used artificial intelligence to design flexible learning environments that integrate students’ cultural identity with scientific understanding. Students’ lived experiences played a central role in knowledge construction, contributing to increased engagement, richer classroom interaction, and a stronger sense of cultural belonging.

Interview data were consistent with the observations. A large majority of participants (85%; 40 out of 47 teachers) reported that AI-supported interactive learning helps students connect scientific concepts with their cultural identity. One teacher maentioned:

*“AI-based educational applications foster students’ sharing of experiences, like home gardening or water-saving practices, and their connection to science lessons.”*

Another participant expressed:

*“AI creates opportunities for collaborative classroom discussions where students share local examples while learning scientific concepts.”*

A third participant added:

*“When students work in groups on AI-supported projects, I feel they make deeper connections between science and their cultural context.”*

Ingeneral, the findings suggest that AI enhances interactive and collaborative learning by linking scientific concepts to students’ cultural experiences. When integrated with culturally responsive teaching practices, AI-supported activities appear to improve student motivation, deepen conceptual understanding, and promote meaningful learning that connects scientific, social, and cultural dimensions.

### 4.6 Results of Theme Six: Intelligent assessment of cultural values in learning

Classroom observations indicated that 72.3% of participants used AI-supported assessment tools to evaluate students’ understanding of scientific concepts in relation to local cultural contexts. Teachers employed a range of AI-enabled assessment methods, including interactive quizzes, individual tasks, and group projects, all designed to assess both conceptual understanding and cultural integration rather than academic achievement alone. Overall, 38 out of 47 teachers (81%) demonstrated the integration of cultural dimensions into science assessment practices, reflecting a shift toward culturally responsive evaluation approaches. Table 6 presents the main observed classroom activities related to intelligent assessment of cultural values in learning, along with their frequencies and percentages.

**Table 6:** Classroom observation results for intelligent assessment of cultural values in learning.

No.	Instructional Practices	Frequency	Percentage (%)
1	Providing culturally grounded applied tasks	36	76.6
2	Personalized feedback	37	78.7
3	Tracking students’ progress within cultural contexts	35	74.5
4	Incorporating local examples into assessment	34	72.3
5	Providing culturally grounded applied tasks	36	76.6

Table 6 shows that teachers used AI-supported assessment as a broader evaluative approach that incorporates cultural dimensions alongside scientific understanding. Assessment practices extended beyond measuring academic achievement to evaluating how students apply scientific concepts in everyday life and culturally relevant contexts. These practices encouraged deeper learning, critical thinking, and meaningful student engagement.

Interview findings were consistent with classroom observations. Most participants (37 out of 47 teachers; 79%) viewed AI-supported intelligent assessment as an effective tool for promoting cultural identity. One teacher stated:

*“Intelligent assessment lets me understand how learners use scientific explainers to local contexts, for instance the climate effect of agricultural activities.”*

Another participant said:

*“AI assessment tools enable me to provide students with personalized feedback that fosters belonging to the scientific community.”*

A third teacher explained:

*“Intelligent assessment helps in tracking how students understand science in relation to culture, which facilitates cultural identity preservation.”*

Generally, the findings suggest that AI-supported assessment contributes to linking scientific learning with cultural values and provides targeted feedback that supports student understanding and engagement. This approach reflects an emerging shift toward culturally responsive assessment practices in science education.

## 5. Discussion of the results

The findings of this study indicate that the integration of Artificial Intelligence (AI) in science education has the potential to support students’ cultural identity by enabling more adaptive, contextualized, and engaging learning experiences. In particular, AI systems’ ability to tailor instructional materials allowed teachers to provide culturally relevant examples and learning activities, which were reported to enhance students’ understanding and motivation. This effectiveness can be attributed to AI’s capacity to adjust learning content to individual learner needs and to provide scaffolded instructional support. Theoretically, these outcomes align with Vygotsky’s social constructivist theory, which emphasizes the importance of social interaction, scaffolding, and cultural context in the learning process.

However, these positive outcomes should be interpreted with caution, as the evidence is primarily based on teachers’ perceptions and classroom observations. While these qualitative sources offer rich contextual insights, they may also introduce subjectivity and limit generalizability. In addition, the absence of standardized measures of student achievement restricts the ability to draw strong causal inferences regarding the direct impact of AI on learning outcomes. This limitation is consistent with recent empirical research in AI-supported education, which similarly cautions that perceived improvements in engagement do not necessarily translate into measurable academic gains under controlled conditions.

Moreover, the observed improvements in students’ motivation and understanding cannot be attributed exclusively to AI integration. Instead, they are likely influenced by multiple contextual factors, including teacher expertise, instructional design, classroom environment, and students’ prior readiness. This interpretation is consistent with the literature emphasizing that the effectiveness of educational technologies is mediated by pedagogical quality rather than the technology itself.

At the same time, the findings align with UNESCO’s emphasis on culturally meaningful learning experiences and the importance of learners’ cultural contexts in education [57–61]. In this regard, the study contributes to growing evidence suggesting that AI can support culturally responsive and adaptive instructional design when intentionally aligned with local educational and cultural needs. Nevertheless, recent scholarship highlights a critical concern that AI systems may reproduce or amplify cultural bias if training data and instructional inputs are not carefully curated [34,62–65]. This underscores the need for systematic validation and critical oversight of AI-generated educational content.

Within this context, AI appears to function as a mediating tool that supports teachers in integrating cultural signifiers into scientific content, thereby enabling students to connect scientific concepts with their lived experiences. This aligns with inquiry-based learning theory and culturally responsive pedagogy, both of which emphasize contextualized and meaningful learning. However, the effectiveness of such integration depends strongly on teacher mediation and pedagogical competence. Without sufficient AI literacy, cultural integration may remain superficial or risk misrepresentation. This is supported by studies identifying teacher digital competence and AI literacy as key determinants of successful technology integration in education [54,60,66–70].

Furthermore, the results suggest that AI contributes to reshaping instructional practices by promoting interactive, constructivist, and student-centered learning environments. These environments appear to enhance learner engagement through culturally contextualized activities and experiential learning. While this is consistent with emerging literature on AI-supported adaptive learning, caution is warranted, as some longitudinal studies suggest that such improvements may reflect novelty effects rather than sustained pedagogical change [60,64,71–75]. This highlights the importance of long-term evaluation of AI-based interventions.

Similarly, the findings indicate that AI facilitates collaborative and culturally responsive learning environments in which students actively construct knowledge through interaction and problem-solving. Although this aligns with social learning theory [75], it should be noted that such outcomes may also result from teacher-led instructional strategies rather than AI itself. Therefore, AI should be viewed as an enabling tool rather than a direct causal factor in collaborative learning.

In relation to curriculum development, AI was found to support the generation of contextually relevant instructional materials while maintaining alignment with scientific accuracy and cultural integrity. This suggests potential for AI-assisted curriculum innovation when guided by teacher expertise. However, caution is required, as over-reliance on generative AI may lead to content homogenization or reduced epistemic diversity if outputs are not critically reviewed by subject experts [34,60,74,75]. Accordingly, AI should be conceptualized as an assistive co-design tool rather than an autonomous curriculum developer.

In addition, AI-supported collaborative learning encouraged students to share cultural knowledge and relate it to scientific concepts through group-based activities. This finding is consistent with social learning theory [75], which emphasizes learning through interaction and shared meaning-making. However, the extent to which these outcomes are attributable to AI remains uncertain and requires further investigation using more rigorous experimental designs.

Finally, AI-supported assessment practices contributed to evaluating higher-order thinking skills and providing individualized feedback to support deeper learning. Despite these advantages, concerns remain regarding the validity of AI-based assessment systems in capturing culturally embedded reasoning, particularly when algorithms are not trained on diverse cultural datasets. This highlights the importance of human-in-the-loop assessment models to ensure fairness, validity, and cultural sensitivity [63,76,77].

Overall, the findings suggest that AI has the potential to support culturally responsive science education by facilitating contextualized instruction, enhancing interaction, and enabling adaptive assessment. However, these benefits are not inherent to the technology itself; rather, they depend on teacher expertise, instructional design quality, and institutional readiness. Consequently, this study contributes to the ongoing discourse on AI in education by emphasizing the critical role of human mediation and contextual factors in shaping educational effectiveness. It also highlights the need for further mixed-method and longitudinal research to better understand the long-term pedagogical impact of AI integration in culturally diverse classrooms.

## 6. Conclusions, recommendations, limitations, and future research directions

The findings of this study suggest that the integration of Artificial Intelligence (AI) in science teaching may contribute to supporting culturally responsive instructional practices and enhancing the integration of cultural identity within science learning contexts. Based on classroom observations and teachers’ perceptions, the results indicate that AI-based tools can assist teachers in selecting culturally relevant materials, adapting instructional content, and designing learning activities that connect scientific concepts with students’ cultural contexts. Collectively, these practices appear to foster more interactive and engaging learning environments, as perceived by participating teachers.

However, it is important to emphasize that these findings do not establish causal relationships between AI integration and student achievement, as the study relied exclusively on qualitative data without standardized or quantitative measures of learning outcomes. Accordingly, the reported improvements in engagement and understanding should be interpreted as context-specific perceptions rather than generalizable or measurable effects. Within this framework, AI is better conceptualized as a pedagogical support tool that facilitates scaffolded instruction, formative feedback, and culturally contextualized learning experiences, rather than replacing teachers’ instructional decision-making.

In this regard, the study contributes to the growing body of literature on Artificial Intelligence in education by offering qualitative insights from experienced teachers on the perceived pedagogical value of AI in culturally responsive science teaching. In particular, it highlights how AI tools are perceived to support the integration of local cultural knowledge into science curricula, enhance the design of interactive and collaborative learning activities, and strengthen formative and adaptive assessment practices. It also underscores the central role of teacher agency in mediating AI integration within culturally grounded instructional contexts, thereby contributing to the limited empirical research at the intersection of AI technologies, cultural pedagogy, and instructional design.

From a pedagogical perspective, the findings suggest several implications. These include the importance of integrating local cultural resources into science instruction to enhance relevance and engagement, the need to design collaborative and student-centered learning environments supported by AI tools, and the potential of AI-assisted formative assessment in informing instructional decisions. Additionally, the findings highlight the critical importance of teacher professional development in AI literacy to ensure effective pedagogical integration. These implications should be interpreted as practice-informed recommendations derived from qualitative evidence rather than empirically validated causal effects on student outcomes.

Nevertheless, several limitations should be acknowledged. The sample consisted of 47 experienced and “distinguished” teachers from the Qassim and Al-Ahsa regions, selected through purposive sampling. While this approach enhanced the depth and richness of the data, it may also introduce selection bias, as participants are likely to possess higher pedagogical expertise and greater familiarity with innovative instructional practices, including AI integration, compared to the broader teacher population. Therefore, the findings should be interpreted within this specific context and may not be fully generalizable to other educational settings.

In addition, the study relied exclusively on qualitative methods, including semi-structured interviews and classroom observations. Although these methods provide rich contextual insights, they do not include standardized or objective measures of student learning outcomes, which limits the ability to draw causal inferences regarding the impact of AI on achievement. Furthermore, the findings may be influenced by limitations associated with self-reported data, including subjectivity and social desirability bias, as well as variations in teachers’ prior experience with AI tools.

Building on these limitations, future research is encouraged to include more diverse samples across regions, school types, and levels of teaching experience to enhance external validity. Mixed-methods and experimental designs are also recommended to triangulate qualitative findings with quantitative evidence of student learning outcomes. Longitudinal studies are needed to examine the sustained impact of AI integration on students’ cultural understanding, engagement, and academic achievement over time. Furthermore, future studies should investigate the role of teacher AI literacy and professional development intensity in shaping instructional quality and learning outcomes. Comparative research between AI-supported and non-AI-supported classrooms would also help isolate the specific contribution of AI tools, while emerging technologies such as generative AI, adaptive learning systems, and immersive simulations warrant further investigation in culturally responsive science education.

Overall, this study suggests that AI has potential as a supportive tool for culturally responsive science education when implemented within a clear pedagogical framework and supported by adequately trained teachers. However, the findings remain context-dependent, perception-based, and non-causal. Therefore, they should be interpreted with caution. Ultimately, the effectiveness of AI integration appears to depend more on teacher expertise, instructional design quality, and contextual conditions than on the technology itself.

## Supporting information

S1 Appendix Classroom observation checklist. (DOCX)

S2 Appendix Semi-structured interview schedule. (DOCX)

S3 Appendix Example datasets, including: Example of classroom observation data. Example of interview data. (DOCX)

S4 Appendix Examples of data analysis categories for classroom observations and interviews. (DOCX)
