# Supplementary material for: Enhancing the sustainability of cultural identity in science curricula through artificial intelligence as an innovative educational approach
Source: PLoS One. 2026 Jul 21;21(7):e0353777. doi: 10.1371/journal.pone.0353777 (PMC13387527; doi:10.1371/journal.pone.0353777)
Supplement: S1 Appendix — (DOCX) [file pone.0353777.s001.docx]

**Appendix 1**

**Classroom Observation Checklist**

**A. General Information (Science Classroom Context)**

• School: Anonymized in accordance with ethical guidelines
• Grade Level (Science): Primary Level (Grade 6)
• Date of Observation: 8 September 2025
• Lesson Duration: 45 minutes
• Observer Code: Researcher (Observer A)

**B. Artificial Intelligence Integration in Science Curriculum**

| **Item** | **Indicator** | **Implementation Status** |
| --- | --- | --- |
| 1 | Integration of AI tools in science curriculum delivery |  |
| 2 | Use of AI to enhance understanding of science concepts |  |
| 3 | Use of AI for adaptive/personalized science learning |  |
| 4 | Use of AI to increase student engagement in science learning |  |

**C. Teacher Practices in AI-Enhanced Science Instruction**

| **Item** | **Indicator** | **Implementation Status** |
| --- | --- | --- |
| 5 | Effective use of AI tools to support science teaching strategies |  |
| 6 | Facilitation of inquiry-based science learning using AI |  |
| 7 | Promotion of critical and scientific thinking through AI-supported instruction |  |

**D. Sustainability of Cultural Identity within Science Curriculum**

| **Item** | **Indicator** | **Implementation Status** |
| --- | --- | --- |
| 8 | Linking science concepts to local cultural contexts |  |
| 9 | Integration of cultural values in science instruction |  |
| 10 | Use of AI to reinforce cultural identity in science learning |  |
| 11 | Students’ demonstration of cultural awareness in science learning |  |

**E. Learning Environment in AI-Supported Science Classrooms**

| **Item** | **Indicator** | **Implementation Status** |
| --- | --- | --- |
| 12 | Active student engagement in science learning activities |  |
| 13 | Collaborative learning supported by AI tools |  |
| 14 | Positive and supportive science classroom environment |  |

**F. Integrated Analytical Observation Notes**

(Observer’s analytical notes related to AI integration and cultural identity sustainability in science education)

**G. Overall Observation Summary**

- Key patterns in AI integration in science curriculum
- Evidence of cultural identity sustainability in science learning
- Notable pedagogical practices observed
