# Supplementary material for: Enhancing the sustainability of cultural identity in science curricula through artificial intelligence as an innovative educational approach
Source: PLoS One. 2026 Jul 21;21(7):e0353777. doi: 10.1371/journal.pone.0353777 (PMC13387527; doi:10.1371/journal.pone.0353777)
Supplement: S2 Appendix — (DOCX) [file pone.0353777.s002.docx]

**Appendix 2**

**Semi-Structured Interview Schedule**

**Study Title:**
Enhancing the Sustainability of Cultural Identity in Science Curricula through Artificial Intelligence as an Innovative Educational Approach

**A. General Information**

• Interview Type: Semi-Structured Interview
• Participants: Female Science Teachers
• Estimated Duration: 45–55 minutes
• Data Collection Method: Face-to-face / Online interview (as applicable)
• Purpose of the Interview: To explore teachers’ perceptions and classroom practices regarding the use of Artificial Intelligence (AI) in supporting the sustainability of cultural identity within science education.

**B. Interview Questions**

| **Item** | **Interview Question** |
| --- | --- |
| 1 | How do teachers understand the concept of sustaining cultural identity in the context of AI-enhanced science instruction? |
| 2 | How does the use of artificial intelligence help link scientific concepts with local cultural and environmental values? |
| 3 | What instructional strategies do teachers employ to integrate AI in ways that foster students’ cultural belonging and personalized learning? |
| 4 | How are AI tools incorporated into classroom or extracurricular activities to support cultural values and promote sustainable behavior? |
| 5 | How do teachers evaluate the role of the school in supporting the use of AI to promote the sustainability of cultural identity within science curricula? |

**C. Interviewer Guidelines**

• Probing questions may be used to clarify or deepen participants’ responses when necessary.
• Participants are encouraged to provide concrete classroom examples based on their teaching experience.
• All responses are treated with strict confidentiality and used solely for academic research purposes.
• The interview focuses on teachers’ lived experiences of integrating AI in science education and its role in sustaining cultural identity.
