# Supplementary material for: Enhancing the sustainability of cultural identity in science curricula through artificial intelligence as an innovative educational approach
Source: PLoS One. 2026 Jul 21;21(7):e0353777. doi: 10.1371/journal.pone.0353777 (PMC13387527; doi:10.1371/journal.pone.0353777)
Supplement: S3 Appendix — (DOCX) [file pone.0353777.s003.docx]

# ****Appendix 3****

**(1)**

**Example of classroom observation data**

## ****A. General Information (Science Classroom Context)****

• School: Anonymized in accordance with ethical guidelines
• Grade Level (Science): Primary Level (Grade 6)
• Date of Observation: 8 September 2025
• Lesson Duration: 45 minutes
• Observer Code: Researcher (Observer A)

## ****B. Artificial Intelligence Integration in Science Curriculum****

| **Item** | **Indicator** | **Implementation Status** | **Examples of Implementation (linked to cultural identity sustainability through AI in science education)** |
| --- | --- | --- | --- |
| 1 | Integration of AI tools in science curriculum delivery | Implemented | AI tools were integrated into science instruction through interactive simulations that connected scientific concepts with students’ local cultural and environmental contexts, supporting cultural identity sustainability. |
| 2 | Use of AI to enhance understanding of science concepts | Implemented | AI-supported explanations and visual models were used to clarify abstract science concepts and link them to culturally familiar real-life examples. |
| 3 | Use of AI for adaptive/personalized science learning | Implemented | AI tools were used to provide differentiated learning support aligned with students’ varying learning needs within culturally relevant science contexts. |
| 4 | Use of AI to increase student engagement in science learning | Implemented | Students engaged with AI-generated simulations and culturally contextualized digital content, which increased motivation and strengthened cultural identity connections. |

## ****C. Teacher Practices in AI-Enhanced Science Instruction****

| **Item** | **Indicator** | **Implementation Status** | **Examples of Implementation (linked to cultural identity sustainability through AI in science education)** |
| --- | --- | --- | --- |
| 5 | Effective use of AI tools to support science teaching strategies | Implemented | The teacher used AI-based simulations and instructional tools to support science teaching while embedding culturally relevant examples within explanations. |
| 6 | Facilitation of inquiry-based science learning using AI | Implemented | The teacher guided students through AI-supported inquiry activities that encouraged exploration of scientific concepts within culturally familiar contexts. |
| 7 | Promotion of critical and scientific thinking through AI-supported instruction | Implemented | AI-generated scenarios were used to prompt questioning and discussion, enabling students to develop scientific reasoning linked to real-life cultural contexts. |

## ****D. Sustainability of Cultural Identity within Science Curriculum****

| **Item** | **Indicator** | **Implementation Status** | **Examples of Implementation (linked to cultural identity sustainability through AI in science education)** |
| --- | --- | --- | --- |
| 8 | Linking science concepts to local cultural contexts | Implemented | Science concepts were consistently linked to students’ local environment and cultural practices to enhance cultural identity sustainability. |
| 9 | Integration of cultural values in science instruction | Implemented | Cultural values were embedded in science explanations through teacher-guided discussions supported by AI-generated examples. |
| 10 | Use of AI to reinforce cultural identity in science learning | Implemented | AI tools were used to present contextualized examples that reflected local cultural and environmental realities. |
| 11 | Students’ demonstration of cultural awareness in science learning | Implemented | Students demonstrated awareness of cultural connections when discussing scientific phenomena and real-life applications. |

## ****E. Learning Environment in AI-Supported Science Classrooms****

| **Item** | **Indicator** | **Implementation Status** | **Examples of Implementation (linked to cultural identity sustainability through AI in science education)** |
| --- | --- | --- | --- |
| 12 | Active student engagement in science learning activities | Implemented | Students actively participated in AI-supported activities that integrated scientific learning with culturally relevant contexts. |
| 13 | Collaborative learning supported by AI tools | Implemented | Students worked collaboratively on AI-based tasks that encouraged discussion of both scientific and cultural dimensions. |
| 14 | Positive and supportive science classroom environment | Implemented | The classroom environment was interactive and supportive, fostering engagement in AI-enhanced and culturally responsive learning. |

## ****F. Integrated Analytical Observation Notes****

AI integration functioned as a pedagogical enabler that supported both scientific understanding and cultural identity sustainability. The observation indicated that AI was not used in isolation but was embedded within teacher-mediated instruction that emphasized culturally relevant science learning. The interaction between AI tools, teacher facilitation, and contextual examples contributed to reinforcing students’ connection between scientific knowledge and their cultural environment.

## ****G. Overall Observation Summary****

1. **Key patterns in AI integration in science curriculum:**
   AI was consistently implemented to support simulations, conceptual understanding, and culturally contextualized science learning.
2. **Evidence of cultural identity sustainability in science learning:**
   Strong integration of cultural identity through contextual examples supported by AI-enhanced instruction.
3. **Notable pedagogical practices observed:**
   Inquiry-based learning, collaborative activities, and culturally responsive AI-supported science instruction.

**(2)**

**Example of interview data.**

## ****Semi-Structured Interview (Researcher–Teacher Interview)****

**Study Title:**
Enhancing the Sustainability of Cultural Identity in Science Curricula through Artificial Intelligence as an Innovative Educational Approach

### ****A. General Information****

• Interview Type: Semi-Structured Interview
• Interviewer: Researcher (Observer A)
• Participant: Science Teacher (Previously Observed in Classroom Session)
• Data Collection Method: Face-to-face interview
• Duration: 55 minutes
• Purpose: To explore perceptions and practices regarding the integration of Artificial Intelligence (AI) in science education and its role in supporting the sustainability of cultural identity within science curricula.

### ****B. Interview Questions and Expanded Responses (Respondent Verbatim Narrative)****

**1. How do you understand the concept of sustaining cultural identity in AI-enhanced science instruction?**

“I understand sustaining cultural identity as connecting science content with students’ local culture, values, and lived experiences. Hmm… it is about making science part of students’ identity, not something separate from them. I feel that when science is taught in isolation, students may not relate to it, but when it is linked to their culture and environment, it becomes meaningful. AI supports this by presenting scientific concepts in visual, interactive, and sometimes scenario-based forms that reflect real-life contexts. In this way, students can see themselves within the content, and this strengthens their cultural connection to learning.”

**2. From your classroom experience, how does AI help link scientific concepts with local cultural and environmental values?**

“AI tools such as simulations, digital models, and visual representations make abstract scientific ideas more concrete. Right… when I use these tools, students are able to visualize processes that are normally difficult to explain. For example, in environmental science lessons, I link the content to local climate conditions, water usage, and community practices. Hmm… students react more positively when they recognize familiar situations. They start saying, ‘this is like what we see in our area,’ and this shows that the scientific concept has become connected to their cultural and environmental reality. This connection makes learning deeper and more meaningful.”

**3. What instructional strategies do you use to integrate AI in ways that foster students’ cultural belonging and personalized learning?**

“I rely on guided inquiry, discussion-based learning, and AI-supported visual explanations. I try not to use AI as a presentation tool only, but as a discussion trigger. Hmm… I often pause the AI simulation and ask students to interpret what they see. Then I connect their answers to examples from their daily life and cultural environment. I also try to differentiate my explanations depending on students’ understanding levels. This combination helps students feel that the lesson is designed for them, and it strengthens both engagement and cultural belonging.”

**4. How do you incorporate AI tools into classroom or extracurricular activities to support cultural values and sustainable behavior?**

“I use AI-supported videos, simulations, and interactive tasks during classroom teaching, and sometimes extend these activities to extracurricular learning. Yes… especially in topics related to environment and sustainability. Hmm… I design tasks where students observe AI-generated scenarios and then discuss their impact on the environment and society. For example, they reflect on waste management, water conservation, and local environmental practices. This helps them think critically about their responsibility toward their community. I notice that students become more aware of sustainability when learning is connected to both technology and culture.”

**5. Based on your experience, how does your school support the use of AI to promote cultural identity sustainability within science curricula?**

“The school provides basic technological infrastructure such as smart boards and internet access, and there is encouragement to use digital tools in teaching. Yes, the support is present, but it is mainly technical rather than pedagogical. Hmm… I feel that there is a gap in professional training on how to integrate AI meaningfully with cultural identity in science education. More structured workshops and continuous training would help teachers use AI not just as a tool, but as a pedagogical approach that supports cultural sustainability in learning.”

### ****C. Contextual Note****

This interview was conducted with a science teacher who had previously been observed during an AI-enhanced science lesson. The responses reflect the teacher’s lived classroom experience and were used to triangulate observational data in order to understand how Artificial Intelligence supports both science learning and the sustainability of cultural identity in educational practice.
