# Supplementary material for: Enhancing the sustainability of cultural identity in science curricula through artificial intelligence as an innovative educational approach
Source: PLoS One. 2026 Jul 21;21(7):e0353777. doi: 10.1371/journal.pone.0353777 (PMC13387527; doi:10.1371/journal.pone.0353777)
Supplement: S4 Appendix — (DOCX) [file pone.0353777.s004.docx]

**Appendix 4**

**Examples of data analysis categories for observation and interview**

**1- Analysis of the Results of the First Category**

| **No.** | **Learning Practices** | **Frequency** | **Percentage (%)** | **Teacher Behavior / Use of AI** | **Focus on Cultural Dimension** |
| --- | --- | --- | --- | --- | --- |
| 1 | Integrating physics concepts with the local environment | 46 | 97.9 | Using AI tools to design examples and explain physics concepts related to the desert climate such as heat and dryness and connecting them to everyday phenomena | Presenting scientific content in a way that links concepts to local environmental practices and values, thereby enhancing students’ contextual understanding of science |
| 2 | Linking lessons to familiar cultural practices | 45 | 95.7 | Using AI to generate practical examples related to local agriculture, water conservation, or energy use and incorporating them into interactive class activities | Making science lessons part of students’ everyday cultural experiences so they perceive science as connected to their cultural and practical lives |
| 3 | Adapting examples to students’ varying levels | 44 | 93.6 | Using intelligent systems to suggest scientific examples and experiments appropriate for different levels of understanding within the classroom | Enhancing cultural belonging by using familiar local examples tailored to each group, supporting both individual and collaborative engagement |
| 4 | Using AI tools to explain scientific experiments | 42 | 89.4 | Designing simplified scientific experiments using AI tools for example, simulating local soil conditions or the effect of sunlight on the environment and presenting results interactively | Linking scientific experiments to local customs and environmental experiences, making scientific understanding more culturally grounded |
| 5 | Encouraging students to provide local examples | 40 | 85.1 | Using AI to generate interactive questions and activities that prompt students to share examples from their own environment and daily practices | Strengthening meaningful learning by integrating scientific knowledge with everyday life experiences and supporting cultural identity and belonging |

A considerable majority of participants (91%) asserted that AI smart personalization of educational content sustains the cultural identity embedding local cultural elements and day-to-day experiences in learning scientific concepts. One participant stated:

*“AI tools help me present scientific content in a way that aligns with the Saudi environment. When I connect physics concepts to the desert climate or the nature of the land, the lesson becomes more accessible to students.”*

This illustrates role of artificial intelligence and how Saudi educators perceive, and the system sustains the cultural identity of the learners by facilitating the integration of local cultural elements. Another participant said:

*“Being able to use AI-supported intelligent personalization means that I can connect science lessons to readily identifiable practices like farming or water saving, which makes students feel that science is embedded in their culture.”*

This illustrates that the blending of scientific knowledge and understanding and cultural knowledge is the main way to enable learning and, at the same time, preserve the cultural identity of the learner. Another participant stated:

*“When the intelligent system suggests local examples that match students’ levels, I notice a clear improvement in engagement and comprehension.”*

**2- Analysis of the Results of the Second Category**

| **No.** | **Instructional Practices** | **Frequency** | **Percentage (%)** | **Teacher Behavior / Use of AI** | **Focus on Cultural Dimension** |
| --- | --- | --- | --- | --- | --- |
| 1 | Linking heritage stories to scientific concepts | 40 | 85.1 | Using AI to select heritage stories and traditional practices and connecting them interactively to scientific concepts | Integrating scientific knowledge with local cultural values to deepen students’ understanding and connect science to their identity |
| 2 | Extracting environmental values from local heritage | 38 | 80.8 | Using AI tools to analyze heritage texts, extract environmental values, and link them to environmental science lessons | Enhancing environmental awareness by connecting scientific learning with local values and conservation practices |
| 3 | Integrating traditional proverbs with scientific experiments | 37 | 78.7 | Employing AI to generate examples drawn from traditional practices and linking them to scientific concepts during classroom experiments | Increasing the relevance and cultural resonance of scientific learning |
| 4 | Analyzing heritage texts to extract scientific principles | 36 | 76.6 | Using AI to identify scientific principles embedded in heritage texts and linking them to the curriculum | Presenting science within a culturally familiar frame to facilitate comprehension |
| 5 | Enhancing discussions on cultural practices and science | 35 | 74.5 | Using AI to generate discussion questions derived from local cultural sources | Strengthening interactive learning and activating cultural identity through shared ideas and experiences |

The data from the interviews and the classroom observations align perfectly. Of the study’s participants, 85.1% (40 out of the 47) stated that teaching with AI while analyzing cultural texts and resources is a constructive pedagogical practice for sustaining cultural identity in the teaching of the sciences. One teacher stated:

*“AI assists me in using heritage stories and traditional practices, integrating them with science lessons, and allowing students to perceive the curriculum as culturally relevant to them.”*

This statement highlights the effectiveness of utilizing cultural resources to aid the understanding of relevant science concepts in students lived experiences. One participant similarly noted:

*“Using AI tools, I can extract environmental values from local heritage and relate them to environmental science lessons, such as conserving natural resources.”*

This demonstrates the ability of AI to culturally contextualize and, subsequently, achieve scientific learning goals. Similarly, one participant voiced:

*“Integrating traditional proverbs or practices with scientific concepts, supported by AI, make learning more meaningful and impactful.”*

**3- Analysis of the Results of the Third Category**

| **No.** | **Instructional Practices** | **Frequency** | **Percentage (%)** | **Teacher Behavior / Use of AI** | **Focus on Cultural Dimension** |
| --- | --- | --- | --- | --- | --- |
| 1 | Designing hands-on experiments linked to the local environment | 42 | 89.4 | Using AI to generate scientific experiments simulating the local environment (the impact of sunlight in desert climates, or the properties of local soil) | Connecting scientific experiments to local traditions and practices to deepen scientific understanding in students’ cultural context |
| 2 | Organizing interactive competitions based on cultural elements | 40 | 85.1 | Employing AI to create competition questions and activities incorporating proverbs, local practices, or cultural symbols | Enhancing student engagement with cultural and scientific content simultaneously |
| 3 | Using educational games to simplify scientific concepts | 39 | 82.9 | Using AI tools to design digital games that connect scientific concepts to local values | Merging scientific knowledge with cultural experiences and recreational activities to improve understanding and engagement |
| 4 | Encouraging students to suggest heritage-inspired solutions | 38 | 80.8 | Using AI to generate scenarios and questions that prompt students to propose scientific solutions based on local heritage | Fostering critical thinking and innovation while maintaining cultural identity |
| 5 | Incorporating interactive technologies into classroom presentations | 37 | 78.7 | Using AI to prepare interactive presentations that link scientific concepts to local traditions | Presenting science through a familiar cultural lens to facilitate comprehension and participation |
| 6 | Using culturally grounded digital storytelling | 35 | 74.5 | Utilizing AI to create digital stories combining scientific concepts with local cultural narratives | Enhancing story-based learning and connecting scientific knowledge to cultural identity and heritage |
| 7 | Customizing learning activities based on students’ cultural backgrounds | 33 | 70.2 | Using AI to analyze students’ interests and suggest activities that align with their cultural backgrounds | Supporting inclusive learning through respect for cultural diversity within the classroom |

89.4% interviewees consider the design of AI supported interactive learning activities as important for the maintenance of cultural identity. One participant said:

*“AI helps me create local heritage-focused activities and educational activities, which help students engage with scientific concepts.“*

Another participant said:

*"Intelligent tools help me create educational games so students can bridge cultural values and scientific concepts. It's so much fun."*

**4- Analysis of the Results of the Fourth Category**

| **No.** | **Instructional Practices** | **Frequency** | **Percentage (%)** | **Teacher Behavior / Use of AI** | **Focus on Cultural Dimension** |
| --- | --- | --- | --- | --- | --- |
| 1 | Developing instructional units aligned with community context | 41 | 87.2 | Using AI to design units on topics such as renewable energy and water conservation | Connecting scientific content with local values and practices to deepen understanding and reinforce cultural identity |
| 2 | Updating scientific content while preserving cultural relevance | 39 | 82.9 | Using AI tools to review and update scientific curricula in line with recent scientific advancements | Ensuring the continuation of cultural identity within the curriculum without compromising scientific modernity |
| 3 | Designing learning activities reflecting local traditions | 38 | 80.8 | Using AI to generate activities linking scientific concepts with local customs and traditions | Strengthening students’ connection to science through culturally familiar activities |
| 4 | Proposing community-related applied topics | 37 | 78.7 | Using AI to provide examples and practical projects grounded in the local environment | Supporting learner-centered education while linking it to cultural identity |
| 5 | Designing culturally contextualized assessments | 36 | 76.6 | Using AI to develop assessment questions that account for students’ cultural backgrounds | Assessing learning in a culturally sensitive manner that reinforces cultural belonging |

Most interviewees (38 of 47 teachers; 81%) stated that the use of AI for curriculum development achieves a reconciliation of the updating of scientific information with the retention of incorporation of cultural information. One teacher said:

*“AI assists in constructing instructional units that serve the sociocultural realities of Saudi Arabia such as renewable energy and water conservation.”*

Another said,

*“AI in curriculum development ensures that information will be up to date, and the cultural dimension will not be lost.”*

A third one said,

*“When the curriculum culturally represents the society it serves, it has more value and impact for the learners.”*

**5- Analysis of the Results of the Fifth Category**

| **No.** | **Instructional Practices** | **Frequency** | **Percentage (%)** | **Teacher Behavior / Use of AI** | **Focus on Cultural Dimension** |
| --- | --- | --- | --- | --- | --- |
| 1 | Enhancing group and interactive discussions | 41 | 87.2 | Using AI applications to generate discussion prompts and classroom activities that encourage group dialogue | Linking discussions to daily practices and local values, such as home gardening and water conservation |
| 2 | Implementing AI-supported group projects | 39 | 83.0 | Using intelligent tools to organize students into groups and manage projects that connect science with everyday life | Encouraging students to relate scientific concepts to their cultural traditions and community practices |
| 3 | Sharing examples from the local environment | 40 | 85.1 | Using AI to generate activities that allow students to share examples from their daily lives | Integrating everyday experiences into scientific learning to enhance meaning and understanding |
| 4 | Organizing interactive educational competitions | 38 | 80.8 | Using intelligent systems to create competitions and interactive questions that stimulate engagement | Linking competitions to local customs, environmental values, and cultural traditions |
| 5 | Group problem-solving activities | 36 | 76.6 | Presenting applied scenarios through AI for solving scientific problems within groups | Encouraging critical thinking and connecting solutions to the local cultural context |

85% (40 of 47 interviewees) stated AI-enabled interactive learning assists students in integrating scientific understanding and cultural identity. One teacher stated:

*“AI-based educational applications foster students' sharing of experiences, like home gardening or water-saving practices, and their connection to science lessons.”*

Another said:

*“AI creates opportunities for collaborative classroom discussions where students share local examples while learning scientific concepts.”*

A third participant stated:

*“When students work in groups on AI-supported projects, I feel they make deeper connections between science and their cultural context.”*

**6- Analysis of the Results of the Sixth Category**

| **No.** | **Instructional Practices** | **Frequency** | **Percentage (%)** | **Teacher Behavior / Use of AI** | **Focus on Cultural Dimension** |
| --- | --- | --- | --- | --- | --- |
| 1 | Providing culturally grounded applied tasks | 36 | 76.6 | Using AI tools to design interactive tests that measure students’ ability to relate scientific concepts to local cultural values | Assessing students’ understanding of scientific phenomena within familiar cultural contexts, such as the impact of climate on agriculture |
| 2 | Personalized feedback | 37 | 78.7 | Designing hands-on activities through AI to apply scientific concepts to local real-life situations | Integrating assessment with students’ everyday experiences to reinforce cultural identity |
| 3 | Tracking students’ progress within cultural contexts | 35 | 74.5 | Using AI to offer individualized feedback that strengthens the connection between scientific knowledge and cultural values | Supporting deep learning and encouraging critical thinking about the relationship between science and society |
| 4 | Incorporating local examples into assessment | 34 | 72.3 | Relying on intelligent assessment tools to track students’ conceptual development and align their performance with cultural standards | Promoting sustainable cultural identity by monitoring scientific understanding within the local cultural framework |
| 5 | Providing culturally grounded applied tasks | 36 | 76.6 | Integrating local examples and traditional practices into activities and interactive assessments | Making assessment a meaningful part of both cultural and scientific learning |

Most interview results aligned with what was observed in the classroom: most of the interviewees (37 out of 47 teachers or 79% of them) regarded AI-assisted intelligent assessment as a means of promoting cultural identity.

One teacher said:

*"Intelligent assessment lets me understand how learners use scientific explainers to local contexts, for instance the climate effect of agricultural activities.”*

Another teacher said:

*"AI assessment tools enable me to provide students with personalized feedback that fosters belonging to the scientific community."*

A third teacher stated:

*“Intelligent assessment helps in tracking how students understand science in relation to culture, which facilitates cultural identity preservation.”*
